# A Picomolar MBP-Binding Nanobody for Target Enrichment and Modular Complex Engineering

**DOI:** 10.1021/acsbiomedchemau.6c00053

**Published:** 2026-07-06

**Authors:** Monica Chinellato, Elisa Franzin, Filippo Vascon, Paul-Albert Koenig, Florian I. Schmidt, Patrizia Pontisso, Laura Cendron

**Affiliations:** † Department of Medicine, 9308 University of Padova, Via Giustiniani n.2, 35128 Padova (PD), Italy; ‡ Department of Biology, University of Padova, Via U. Bassi n.58/b, 35121 Padova (PD), Italy; § Core Facility Nanobodies, University Hospital Bonn, Biomedical Center (BMZ 1), 202 Venusberg-Campus 1, 53127 Bonn, Germany; ∥ Institute of Innate Immunity, University Hospital Bonn, Biomedical Center (BMZ 2), 2G Venusberg-Campus 1, 53127 Bonn, Germany

**Keywords:** Single-domain antibodies, heavy chain only antibodies, Maltose-Maltodextrin Binding Protein, yeast surface display, protein complexes engineering, macromolecules purification, CryoEM

## Abstract

The variable domains of heavy chain only antibodies, also called nanobodies, have provided powerful new tools for biomedical research. Their rapid development across multiple fields has helped address emerging scientific and clinical challenges, establishing single-domain antibodies (sdAbs) as highly adaptable platforms for a wide range of biotechnological and therapeutic applications. In this study, we identified a nanobody from an alpaca immune library, sdAbCM1, that binds to the maltose-maltodextrin binding protein, MBP, with picomolar affinity. Biophysical investigations showed that sdAbCM1 recognizes a yet unreported epitope and interacts with MBP in its closed conformation when maltose is present. The high affinity, epitope specificity, and ability to tolerate the ligand-bound state render sdAbCM1 a valuable and versatile biotechnological tool with potential applications such as detection, localization, and purification of MBP fusion proteins and protein complex engineering for single-particle microscopy.

## Graphical abstract

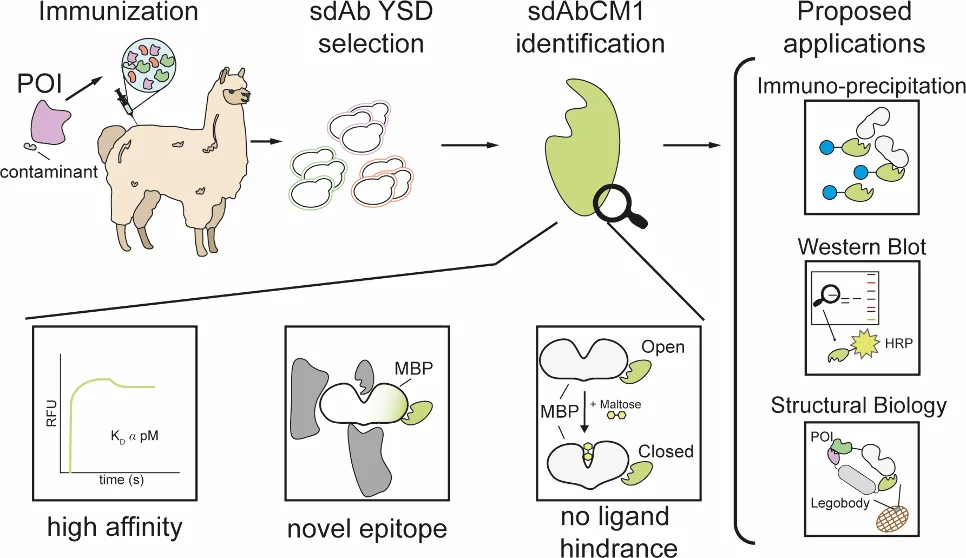

Antibody discovery is a cornerstone of modern medicine, paving the way for more specific and sophisticated theragnostic strategies. To address emerging biomedical challenges, it has become essential to accelerate discovery pipelines. Traditionally, antibodies were developed by small animal immunization and followed by hybridoma generation.^1,2^ The generation of libraries of antibody fragments or antibody-like proteins and the invention of phage display helped accelerate the discovery process and provided remarkable versatility. Precise genetic manipulation and protein engineering further enabled the isolation and generation of functional antibodies in a short time.^3,4^

More recently, yeast surface display (YSD) was described as an alternative technology for the discovery of antigen-binding proteins.^5^ While library size and overall throughput is smaller than for phage display,^6^ YSD provides multiple advantages, including improved synthesis of antibody fragments in a eukaryotic context and more versatile options for selection by fluorescence-activated cell sorting (FACS). YSD also enables the precharacterization by flow cytometry without the need for antibody purification.^7,8^ One more transformative event for display technologies in the pharmaceutical field has been the discovery of heavy chain only antibodies in camelids. The variable domain of heavy chain only antibodies (VHHs) can be expressed as stand-alone single-domain antibodies (sdAbs), also referred to as nanobodies.^9^ They can be obtained by camelid immunization without requiring animal sacrifice and yield approximately 10^7^–10^8^ unique clones after immunization.^10,11^ Their small size, stability, and unique epitope recognition properties make them a game changer for theranostics and biotechnological applications. Here we describe the serendipitous discovery of a unique sdAb against MBP and characterize it for its properties and potential applications. Briefly, an alpaca was immunized with a cocktail of antigens to enhance immune response, following standard procedures. Nanobody coding sequences were obtained from lymphocytes and used to construct a yeast display library (see Supporting Information Methods). The library was screened against purified biotinylated SERPINB3, recombinantly produced in *E. coli* as an untagged protein present in the immunization cocktail (Figure 1a). The latter is a member of the serin protease inhibitor family, selected due to its role in modulating cell survival and its prognostic role in solid tumors.^12–14^

**Figure 1:**
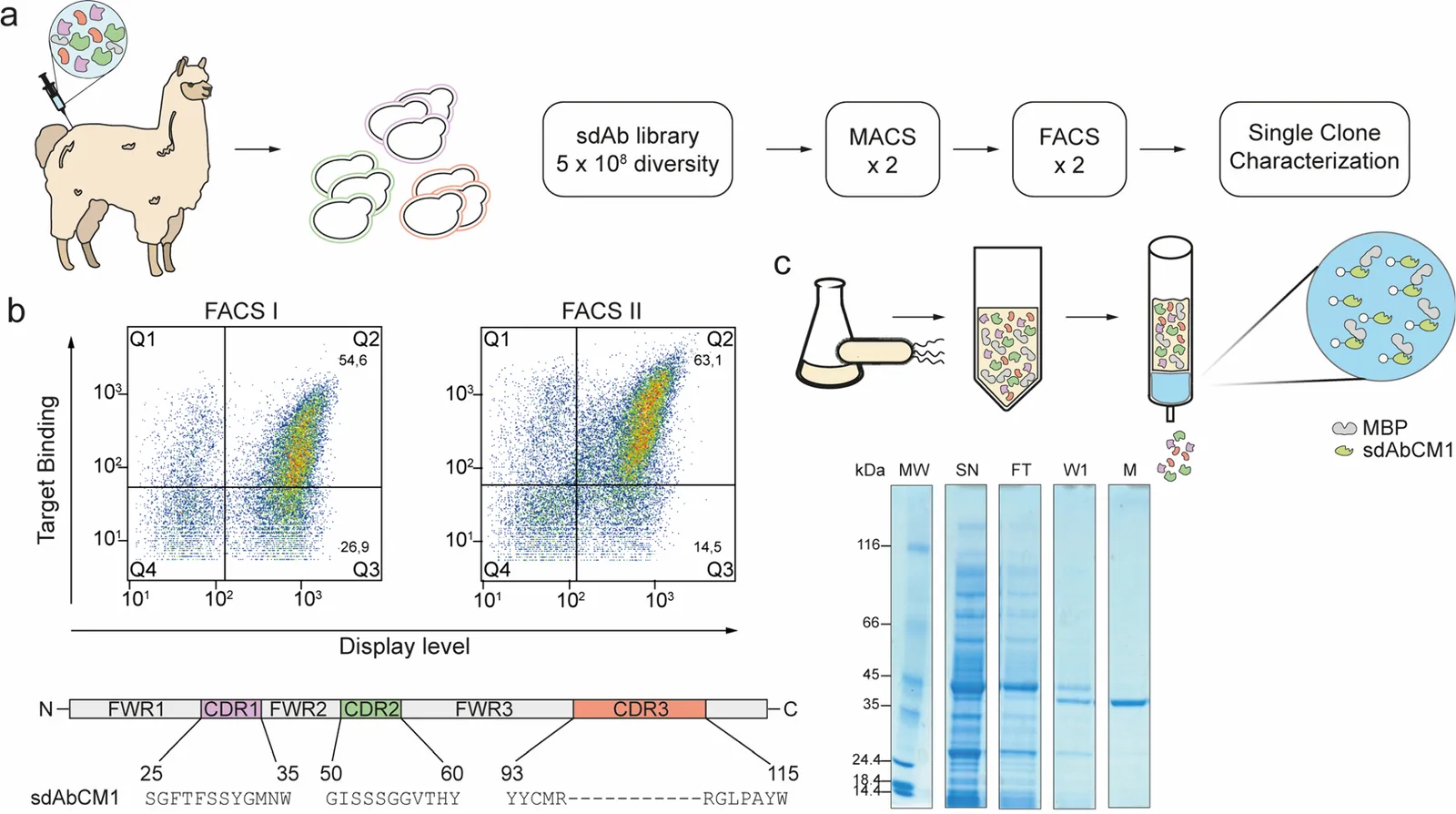
Nanobody library generation and protein sample identification. (a) Process scheme: alpaca immunization, yeast cells displaying the library on their surface and screening steps. (b) Density plot of FACS selection performed on the YSD library and schematic representation of sdAbCM1’s CDRs (c) Graphical representation of the purification with a sdAbCM1-functionalized resin on a crude *E. coli* lysate, underneath the Coomassie-stained SDS-PAGE of each chromatographic step (SN: crude lysate, FT: unbound fraction, W1: wash, M: eluted MBP). The graphics and schemes in the figure were created using Biorender and Microsoft Powerpoint tools.

After three rounds of FACS selection (Figure 1b), a few clones were identified and preliminarily characterized by flow cytometry to evaluate the apparent binding affinity (K_D_^app^) for SERPINB3.

One nanobody candidate, sdAbCM1, was chosen for recombinant expression, since the other ones presented extra noncanonical cysteine residues in the CDRs (Table S1). Although the yeast display data suggested nanomolar affinity, sdAbCM1 showed inconsistent binding across different preparations of purified SERPINB3 antigen in the ELISA assays (Figure S1a–c). SDS-PAGE analysis of the antigen preparation revealed two closely migrating bands (Figure S1d), initially attributed to the already described tendency of SERPINB3 to be processed by proteases.^15–17^ An accurate analysis of the recombinant samples by LC–MS/MS (EMBL proteomics facility) revealed that the upper band was correctly attributed to recombinant SERPINB3, while the lower band was identified as *E. coli* endogenous MalE (UniProt: P0AEY0), also known as Maltose-Maltodextrin Binding Protein (MBP; Figure S1e,f, Table S2).

To explore sdAbCM1’s ability to capture MBP, a crude *E. coli* lysate was tested in an immunoprecipitation experiment. sdAbCM1 was covalently coupled to an agarose resin, loaded with *E. coli* lysate (see Supporting Information and Methods), washed extensively and the retained protein was eluted under acidic conditions, yielding a single band at 42 kDa (Figure 1c). This assay demonstrated that sdAbCM1 specifically binds MBP, a bacterial contaminant, rather than the initially desired human target SERPINB3. Yet, because MBP is extensively used both as a fusion partner to enhance solubility and as a moiety to enlarge or stabilize protein complexes for Cryo-EM studies,^18–20^ we decided to further characterize sdAbCM1 as a potential reagent for target detection, localization, enrichment, and structural applications.

Several groups have reported the discovery of MBP binders, mainly with the scope of validating synthetic libraries or finding novel probes for biophysical studies. These binders were identified from libraries of synthetic antibodies or antibody fragments (e.g., sAB-M11, Sb_MBP1, Sb_MBP2, Sb_MBP3)^21,22^ and alternative binding scaffolds, such as ankyrin domains (off7, mbp3_5, mbp3_16).^23^

To characterize sdAbCM1, we first determined the structure of the complex with MBP by X-ray crystallography. The high-resolution structure (1.75 Å; PDB 9TY2, Table S2) revealed that sdAbCM1 binds the N-terminal domain of MBP (residues 1–109 and 264–309) without interfering with maltose binding rearrangements (Figure 2a). In the crystal lattice, MBP adopts its closed conformation, with a disaccharide molecule bound in the canonical pocket. The maltose molecule is stabilized by Trp63 and Tyr156 (Figure 2b,c), and the closed conformation is maintained through hinge-region rearrangements (residues 110–113, 259–263, and 310–315), consistent with prior observations.^25^ MBP-sdAbCM1 interface involves the nanobody CDRs (651 Å^2^ buried surface area) contacting a major surface of the MBP N-terminal domain (644 Å^2^ buried area) (Figures 2a,b,d).

**Figure 2:**
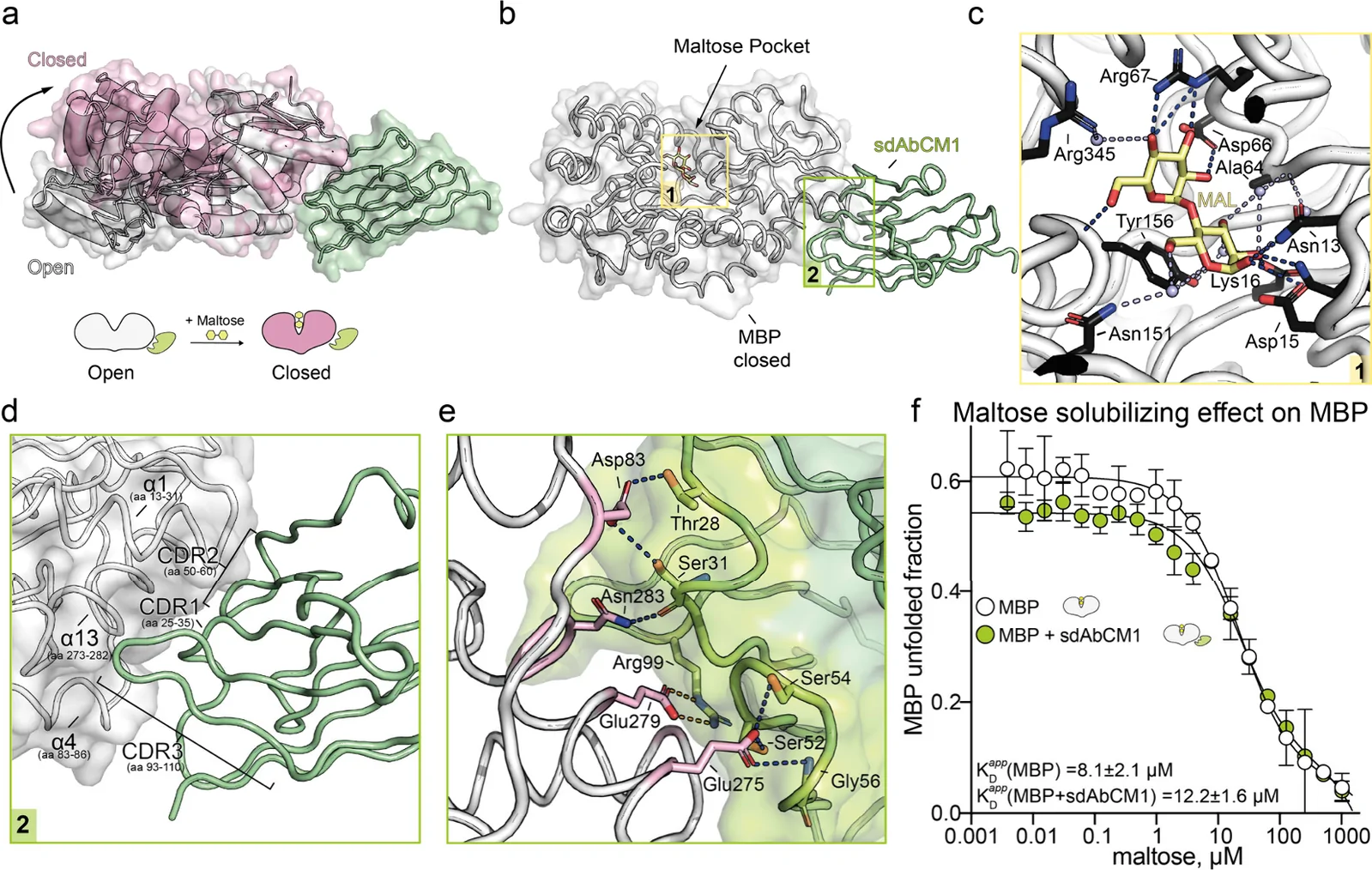
Structural determinants of sdAbCM1 binding site on MBP N-terminal domain and impact of maltose binding properties. (a) Superimposition of open and closed MMBP based on sdAbCM1 epitope, with a cartoon showing the conformational change upon maltose binding. (b) General view of MBP-sdAbCM1 complex. (c) Zoom on the maltose binding site, maltose (yellow) is coordinated by residues indicated in black, water molecules are in pale blue. (d) Zoomed view on the MBP epitope (white) bound by sdAbCM1’s CDRs (pale green). (e) Details of the main residues involved in the interaction between sdAbCM1 (pale green) and residues of the NTD of MBP (baby pink), surface in lime represent sdAbCM1 CDR. (f) Isothermal analysis of Thermofluor data to determine the impact of sdAbCM1 to the MBP-Maltose affinity. The fraction of unfolded MBP at *T*_m_ + 1 °C, with or without sdAbCM1, is plotted as a function of maltose concentrations. K_D_^app^ values were calculated from the EC_50_ as previously reported by Bai et al.^24^ Interactions are represented by dashed lines as follows; hydrogen bonds are in blue and salt bridges in yellow.

In more detail, CDR1 and CDR2 establish multiple hydrogen bonds with the MBP α-helices α1, α4 and α13, involving mainly the residues 83–86 and 273–285. CDR3 extends further toward helix α13, making contacts with residues Asn283, Tyr284, and Thr287. The complex is further stabilized by a salt bridge between Arg99 of sdAbCM1 and Glu279 of MBP (Figure 2e, Figure S2).

To confirm that sdAbCM1 did not hinder sugar binding by MBP, thermal shift assays exploring the thermostabilizing effect of maltose were performed both in the presence and the absence of sdAbCM1, equimolar to MBP (Figure 2f; Figure S3). Consistent with the crystallographic observations, the presence of the nanobody did not affect MBP’s affinity to maltose.

In other reported cases, the binding affinity of synthetic sdAbs or Fabs to MBP was influenced by conformational changes associated with maltose binding. For example, the family of synthetic nanobodies Sb_MBP1–3, which bind the cleft between the N- and C-terminal domains of MBP, revealed a 58-fold decrease in sdAbs’ affinities at maltose-saturating concentrations in surface plasmon resonance (SPR) analysis.^22^ In contrast, sAB-11M, which was used to measure MBP conformational dynamics, binds the hinge region of MBP, stabilizing the closed conformation. It exhibits strong positive cooperativity with maltose, enhancing the affinity by ∼100-fold in the presence of the disaccharide.^21^

To define the thermodynamic and kinetic determinants of complex formation, recombinant MBP was immobilized on a CM5 sensor chip, and sdAbCM1 was injected at concentrations ranging from 0.04 to 30 nM. The fast association and slow dissociation rates yielded an affinity of 45.8 ± 0.9 pM (Figure 3a). Compared with literature values for other MBP binders, this affinity establishes sdAbCM1 as the stronger binder reported for MBP (Figure 3b), exceeding by nearly 2 orders of magnitude the affinity of sAB-11 M in the presence of maltose.

**Figure 3:**
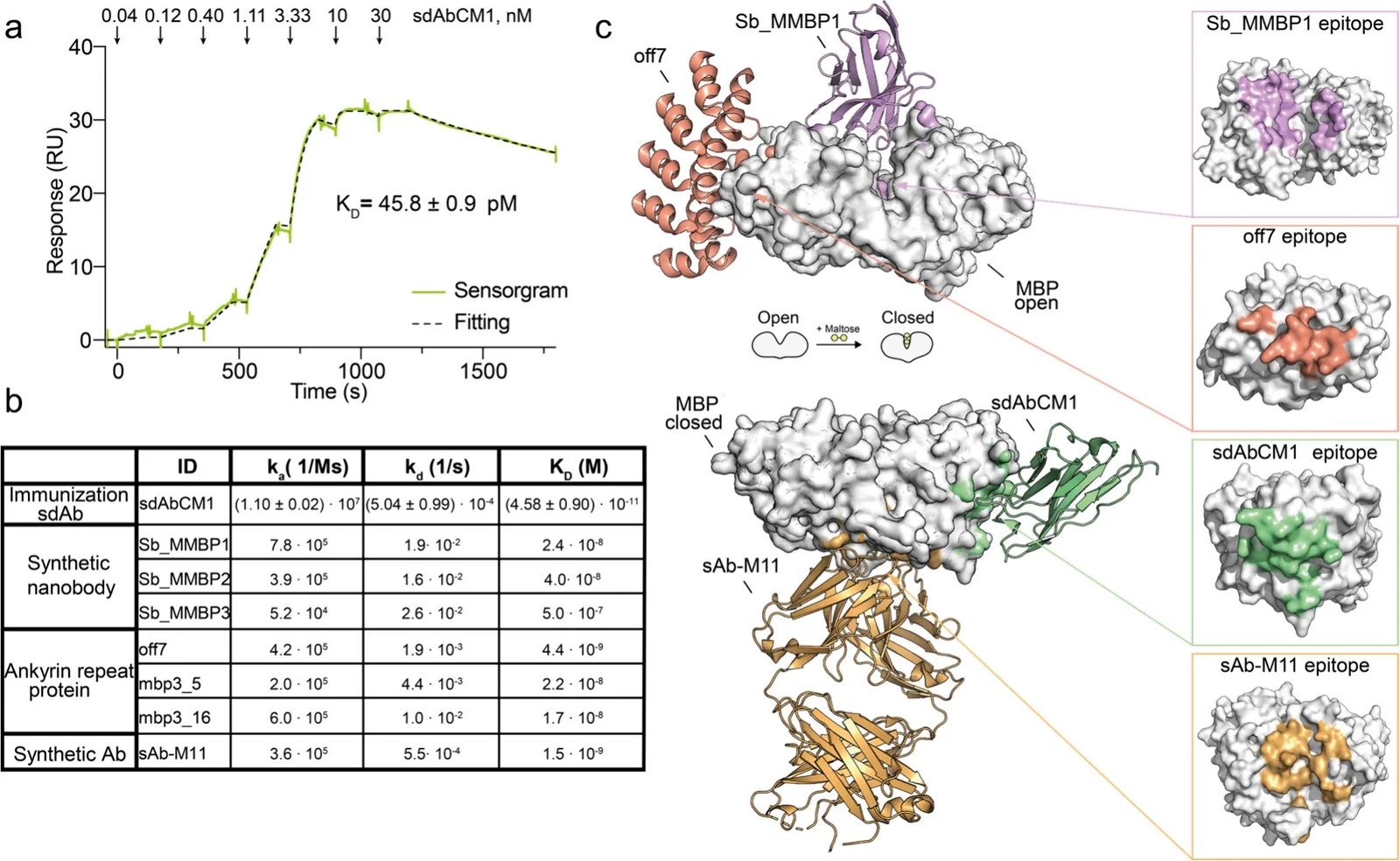
Comparison of affinity, binding site and interaction surface of different binders on MBP. (a) Single cycle SPR sensorgram. Experimental data in green, while kinetic fitting in dashed black. (b) Table comparing kinetic binding data obtained by SPR for sdAbCM1 and synthetic nanobodies, antibodies and ankyrin repeat proteins reported in literature. Data presented without error are presented as their respective publications. (c) Frontal view of MBP (white) in open and closed conformation, respectively with their binders: open with Sb_MBP1 (PDB ID 5M13, in lilac) and off7 (PDB ID 1SVX, in pale orange); closed with sdAbCM1 (pale green) and sAb-M11 (PDB ID 5BJZ, in gold). Recognized epitope on MBP is reported with each respective color in zoomed boxes.

To further evaluate sdAbCM1’s properties, superimposition of the sdAbCM1–MBP complex onto all structurally characterized MBP binders (Figure 3c) revealed that sdAbCM1 engages a unique binding surface, independent of MBP conformation, making sdAbCM1 fully complementary to previously reported MBP binders.

Given sdAbCM1’s picomolar affinity and the tendency of nanobodies to target conformational epitopes, we tested whether sdAbCM1 could recognize MBP after electrophoresis under denaturing conditions, followed by nitrocellulose transfer (Western blotting) as well as potential cross-reactivity in eukaryotic cell lysates. As reported in Figure S4, MBP is clearly detected by sdAbCM1 down to 25 ng loaded per gel lane, whether MBP is isolated, present in an *E. coli* lysate, or fused to a protein of interest; no cross-reactivity was detected in a HEK293 cell lysate.

In this short report, we describe the serendipitous discovery and detailed characterization of sdAbCM1, a nanobody selected from an alpaca immune library. Structural studies reveal that sdAbCM1 binds a previously unreported epitope on the N-terminal domain of MBP without perturbing maltose binding. Thermal shift assays indicate that sdAbCM1 does not disrupt MBP’s maltose-binding properties. Surface plasmon resonance analyses show binding in the low-picomolar range, among the tightest MBP binders described to date, and Western blot (WB) experiments demonstrate sdAbCM1’s ability to detect MBP in crude, native, and denatured samples with high sensitivity and specificity. Taken together, these unique properties make sdAbCM1 a versatile all-in-one tool for biochemical, biophysical, and cellular biology assays. The scientific community can exploit it to design fusion constructs tailored to specific downstream applications, such as enrichment of protein complexes by immunoprecipitation, detection and immobilization of MBP fusions on functionalized surfaces, immunofluorescence assays, and clean removal of MBP from proteolyzed fusion constructs, implementing standard amylose purifications (Figure S5). Importantly, as demonstrated above, sdAbCM1 shows full compatibility with traditional MBP purifications, confirming that MBP-sdAbCM1 complex formation does not interfere with classical affinity purification workflows. Furthermore, MBP-sdAbCM1 complex formation could contribute to the stabilization of protein complexes, analogous to SpyTag/SpyCatcher approaches.^26,27^

Of particular note is the suitability of sdAbCM1 for structural applications, in particular, Cryo-EM. As megabodies have been assessed as tools to stiffen and enlarge proteins for particle picking,^28^ sdAbCM1 reformatted as a megabody construct could be beneficial for particle enlargement while still maintaining high resolution of the distal portion of the protein of interest. Incorporating sdAbCM1 into modular tools such as legobodies^29^ could offer even greater efficiency, as their design allows the simultaneous use of multiple binding blocks to stabilize the complex of interest, including transient ones, potentially leading to improved particle distributions and higher resolutions.

These results establish sdAbCM1 as a monomeric and readily accessible biotechnological tool with a strong potential for future applications.

## Supplementary Material



## Notes

Material and methods not included in the main text, sdAb’s CDRs, protein biochemical properties comparison, crystallographic table, sdAbCM1 characteristics and MBP identification, Ligplot, Thermofluor curves, Western blot, sdAbCM1-MBP coelution on amylose resin, and supplementary references (PDF)
