# Supplementary material for: A Picomolar MBP-Binding Nanobody for Target Enrichment and Modular Complex Engineering
Source: ACS Bio Med Chem Au. 2026 Jul 6;6(4):323–8. doi: 10.1021/acsbiomedchemau.6c00053 (PMC13495011; doi:10.1021/acsbiomedchemau.6c00053)
Supplement: Supplementary file 1 [file bg6c00053_si_001.pdf]

A Picomolar MBP-Binding Nanobody for Target Enrichment and Modular Complex Engineering

Authors:

Monica Chinellato[1], Elisa Franzin[2], Filippo Vascon[2], Paul-Albert Koenig[3], Florian I. Schmidt[3,4], Patrizia Pontisso[1], Laura Cendron[2*]

Affiliation:

[1] Department of Medicine, University of Padova, Via Giustiniani n.2, 35128 Padova (PD), Italy

[2] Department of Biology, University of Padova, Via U. Bassi n.58/b, 35121 Padova (PD), Italy

[3] Core Facility Nanobodies, University Hospital Bonn, Biomedical Center (BMZ 1), 202 Venusberg-Campus 1, 53127 Bonn, Germany

[4] Institute of Innate Immunity, University Hospital Bonn, Biomedical Center (BMZ 2), 2G Venusberg-Campus 1, 53127 Bonn, Germany. Bonn, Germany

Supplementary information

Table of content

1. **Materials and methods**
    a. Recombinant proteins expression and purification
    b. Alpaca Immunization and Library generation
    c. Protein preparation and magnetic beads selection
    d. Cell labelling and Fluorescence activated cell sorting (FACS) selection
    e. Single clone identification, molecular cloning
    f. Enzyme-Linked Immunosorbent Assay (ELISA)
    g. Surface Plasmon Resonance
    h. Immunoprecipitation of *E. coli* endogenous MBP
    i. Florescent thermal shift assay
    j. Western Blot
    k. Complex crystallization, data collection, structure determination and analysis
    l. scAbCM1 competition MBP purification on amylose resin

2. **Supplementary figures**
    a. Figure S1: SB3 LC-MS/MS analysis and MBP identification and sdAbCM1 validation
    b. Figure S2: Ligplot map of MBP and sdAbCM1 interacting residues
    c. Figure S3: Normalized Thermofluor curves with maltose titration
    d. Figure S4: Detection on MBP
    e. Figure S5: sdAbCM1 competition over MBP-amylose purification

3. Supplementar**y tables**
    a. Table S1. Nbs CDRs sequences with underlined free cysteine.
    b. Table S2. Chemical properties of protein sequences
    c. Table S3. Crystallographic table

1. **Materials and methods**

a. Recombinant proteins expression and purification

SERPINB3

For alpaca immunization, SERPINB3 was obtained as follows. *E. coli* BL21(DE3) cells (New England Biolabs) were transformed with pET101 coding for SERPINB3, then cultured in LB medium supplemented with ampicillin at 37°C until they reached OD_{600} = 0.4-0.6, when they were induced with 1 mM IPTG. After induction, cells were grown O/N at 16°C and harvested by centrifugation. The pellet was resuspended in Lysis Buffer (25 mM MES, pH 5.0) supplemented with 2x Roche protease inhibitor cocktail and mechanically lysed with One Shot Cell Disruptor (Constant System). The soluble fraction was loaded onto Sepharose SP Fast Flow resin (Cytiva) previously equilibrated in Lysis Buffer. The resin was washed with 2 column volumes (CVs) of Lysis Buffer; step elution was performed with increasing concentrations of Elution Buffer (25 mM MES, 1 M NaCl, pH 5.0). SERPINB3 for selection and biochemical characterization was purified by means of 2 repeated cycles of ion exchange chromatography. Both procedures were followed by Size Exclusion Chromatography performed with Superdex75 26/600 pg using PBS 1x pH 7.4 (Na_2HPO_4 10 mM, KH_2PO_4 1.8 mM, NaCl 137 mM, KCl 2.7 mM) as storage buffer. Protein was concentrated and stored at -80°C.

MBP - Maltose-maltodextrin binding protein

For biochemical and structural characterization MBP protein was produced as follow. *E. coli* BL21(DE3) cells (New England Biolabs) were transformed with pMAL-c2x and were cultured in LB Amp medium until they OD_{600}= 0.4-0.6, when they were induced with 0.2 mM Isopropyl-β-D-1-thiogalactopyranoside (IPTG). After induction, cells were grown O/N at 16°C and harvested by centrifugation. The pellet was resuspended in MBP lysis buffer (20 mM Tris-HCl, 200 mM NaCl, 1 mM EDTA, pH 7.4) supplemented with 1x Roche protease inhibitor cocktail and mechanically lysed with One Shot Cell Disruptor (Constant System). MBP was purified from the soluble fraction by affinity for Amylose Resin (New England Biolabs). The loaded resin was washed with MBP lysis buffer and then the protein was eluted with Buffer B (20 mM Tris-HCl, 200 mM NaCl, 1 mM EDTA, 10 mM Maltose pH 7.4). Size Exclusion Chromatography was performed with Superdex75 26/600 pg using PBS 1x pH7.4 (Na_2HPO_4 10 mM, KH_2PO_4 1.8 mM, NaCl 137 mM, KCl 2.7 mM) as buffer.

sdAbCM1 Nanobody

Electro-competent *Pichia pastoris* (*Komagatella paffii)* SuperMan5 (BioGrammatics) strain was transformed by electroporation with linearized pDNA for genome integration. Cells were initially grown in YPD added with P/S and G418 at 30° C. The cultures were diluted in BMGY, grown for 24 hours, then pelleted and resuspended in BMMY, containing 2% (v/v) methanol, for induction at 30 °C. After 4 days, the supernatant was collected by centrifugation. Nanobody construct, presenting 6-His tag at the C terminus was purified by NiNTA resin. Briefly supernatant was loaded into the resin, extensively washed with Buffer A (KH_2PO_4 28 mM, Na_2HPO_4 32 mM, 500 mM NaCl pH 7.5) and then eluted in Buffer B (Buffer A, 500 mM imidazole). Proteins were then buffer exchanged by size exclusion chromatography (Superdex75 26/600 pg) using PBS 1x pH7.4 (Na_2HPO_4 10 mM, KH_2PO_4 1.8 mM, NaCl 137 mM, KCl 2.7 mM) and stored at -80°C.

b. Alpaca Immunization and Library generation

An alpaca (*Vicugna paco*) was immunized to elicit heavy chain only antibodies against SERPINB3. All immunizations were authorized by the Landesuntersuchungsamt Rheinland-Pfalz and in alignment with European Union animal welfare legislation. Animals were four times injected with 200 µg of SERPINB3 supplemented with GERBU Adjuvant Fama (GERBU Biotechnik GmbH) in two-week intervals. As described by previous protocols (Pardon et al., 2014; Schmidt et al., 2016). Briefly peripheral blood lymphocytes were collected, RNA was extracted and reversely transcribed

to obtain the cDNA of the VHH domain only by means of three sets of primers (random hexamers, oligo(dT), and primers specific for the constant region of the alpaca heavy chain gene).
A yeast surface display (YSD) library was generated following well-established protocols (Angelini et al., 2015), pC-TCON2 plasmid was triple digested with SalI, BamHI, NheI restriction enzymes (New England Biolabs); while VHH coding sequences were amplified by a nested PCR, firstly with degenerated primers (5'-GTCGGCTAGCGCTCAGKTGCAGCTCGTGGAGWCNGG-3' and 5'-TTTTGTTCGGATCCCCGGGAGGAGACGGTGACCTG-3') and then with a couple of primers introducing the homology recombination with the vector sequence (5'-GGAGGCGGTAGCGGAGGCGGAGGGTCGGCTAGC-3' and 5'-GTCCTCTTCAGAAATAAGCTTTTGTTCGGAT-3'). The obtained DNA was used to electroporate electrocompetent cells *S. cerevisiae* RYJ100 (Van Deventer et al., 2015) to cover up to $1x10^9$ clone diversity.

c. Protein preparation and magnetic beads selection

To proceed with binder selection, SERPINB3 was chemically biotinylated with EZ-Link Sulfo-NHS-LC-Biotin kit (Thermo Fisher Scientific) following the manufacturer's instructions, to obtain less than 3 biotin moieties per molecule. Biotinylated SERPINB3 (bSERPINB3) was then subjected to Size Exclusion Chromatography to remove the biotin excess.
For library selection, sufficient glycerol stock to cover the theoretical diversity of the library was thawed in SD-CAA medium supplemented with penicillin/streptomycin (P/S) and cultured overnight at 30°C with shaking. The day after, they were diluted in fresh SD-CAA P/S to OD_{600}= 1 and grown at 30°C until they reached OD_{600}= 2-4. Cells were collected by centrifugation and resuspended in induction media SG-CAA P/S and grown O/N at 20°C. The day after, OD_{600} was measured and cells were collected to cover 10 times the library diversity. Cells were washed twice in PBS pH7.4 added with 0.1% (w/v) Bovine Serum Albumin (PBSA) and incubated with washed Streptavidin conjugated Dynabeads (Thermo Fisher Scientific) for 2 hours. Non-bound cells were collected and subjected again to negative selection. Thereafter, soluble fraction was incubated with magnetic beads that were previously decorated with 50 pmol/$4x10^5$ beads of bSERPINB3. After 2 hours of incubation, magnetic beads were collected, washed 5 times with PBSA and placed in an adequate volume of SD-CAA for overnight growth at 30° C. The day after, magnetic beads were removed and cell collected.

d. Cell labelling and Fluorescence activated cell sorting (FACS) selection

Two rounds of FACS selection were performed as follow. *S. cerevisiae* RJY100 bearing the plasmid coding for VHH antibodies were induced as previously described. $2x10^8$ cells were collected by centrifugation, washed twice with PBSA and resuspended in PBSA containing 1μM bSERPINB3 and the antibody 9E10 against c-myc tag (1:300 dilution; Invitrogen). The incubation was carried out at RT for 1 hour and follow by 2 consecutive washing steps with PBSA. Finally, cells were resuspended in PBSA containing Streptavidin-AlexaFluor 647 and Goat Ig Anti mouse-AlexaFluor 488 (Invitrogen), diluted 200 times. After 30 minutes incubation at 4° C in the dark, cells were washed twice and resuspended in PBSA at $1x10^7$ cells/ml for FACS analysis. Cells were sorted with FACS Aria III (BD Biosciences) and collected in SD-CAA P/S.

e. Single clone identification and molecular subcloning

Cells collected after FACS sorting were grown in SD-CAA P/S media at 30°C, starting from OD= 0.2, until they reached OD=1. $1x10^8$ cells were harvested and subjected to plasmid DNA extraction with Zymoprep Yeast Plasmid Miniprep II (Zymo Research), following manufacturer's instructions. 5 μl of DNA was used to transform chemo-competent *E. coli* TOP10 cells (Thermofisher Scientific). Transformed cells were grown on selective LB plates containing 100 μg/ml ampicillin, overnight at 37°C. Single colonies were selected for plasmid DNA extraction and the obtained material was used for Sanger sequencing to identify the coding sequences. cDNA was amplified by PCR amplification

with Platinum SuperFi II Polymerase (Thermofisher Scientific), using the following primers: GGAGGCGGTAGCGGAGGCGGAGGGTCGGCTAGC and GTCCTCTTCAGAAATAAGCTTTTGTTCGGAT. Amplified cDNA and pJAGαMF vector (Biogrammatics) were double digested with NheI and BamHI (New England Biolabs). Ligation was performed with T4 Ligase (Thermofisher Scientific) following manufacturer's instructions. Colonies positive for the gene insertion were checked by Sanger sequencing.

f. Enzyme-Linked Immunosorbent Assay (ELISA)

sdAbCM1 reactivity against SERPINB3 was assessed by direct ELISA (enzyme-linked immunosorbent assay) assay was performed as described by (Biasiolo et al., 2023) Briefly, 96-well ELISA polystyrene high binding plates (Sigma Aldrich) were coated overnight at 4 °C with 100 µL of SERPINB3 (100 ng/mL) in carbonate buffer pH 9.6. Plates were blocked with PBS pH7.4 added with 5% milk for 2 h at RT, then washed with PBS 0.5% Tween-20 and incubated for 1 h at RT respectively with sdAbCM1(10 µg/ml) and commercially available oligoclonal anti-SERPINB3 antibody (Xeptagen). Washing steps were repeated (6×) and the plates were incubated with 100 µL of mouse anti-His HRP conjugated (1:5000) (clone SB194b, Southern Biotech) for sdAbCM1, and goat anti-rabbit IgG HRP conjugated (1:5000) (A16066, Invitrogen) for oligo anti-SERPINB3. The plates were then washed (6×) and ELISA reaction was initiated by addition of TMB substrate (100 µL). The reaction was quenched by the addition of 1N HCl (100 µL) and the resulting absorbance was measured with a Victor X3 Microplate reader (Perkin Elmer) at 450 nm.

g. Surface Plasmon Resonance

The affinity of CM1 nanobody to MBP was measured by surface plasmon resonance (SPR), using a Biacore 8k (Cytiva) operated with a running buffer containing 20 mM HEPES, 250 mM NaCl, 0.2 mM $CaCl_2$, 0.005% v/v Tween-20, pH 7.0. Purified MBP was diluted to 40µg/mL in 10 mM sodium acetate, pH 4.2 and immobilized on a Series S CM5 sensor chip (Cytiva) via amine coupling, reaching 325 RUs. A flow cell with no immobilized protein was used as a reference. Serial dilutions of the analyte in running buffer were injected over the active and reference flow cells in single-cycle kinetic experiments (30 µL/min flow rate, 120 s of contact time and 600 s of dissociation). Blank runs were performed by injecting only running buffer. Experiments were conducted in duplicate on an independently immobilized ligand. Double referenced sensorgrams were analyzed by the Biacore Insight Evaluation Software, fitting experimental data to a 1:1 binding model and obtaining the steady-state K_D. Further data processing was performed by GraphPad Prism 8.

h. Immunoprecipitation of *E. coli* endogenous MBP

1 ml of NHS-Activated Sepharose 4 Fast Flow (Cytiva) slurry was conjugated with 100 µg of sdAbCM1 following manufacturer's instructions. Resin was then washed extensively to equilibrate in MBP lysis buffer. The supernatant fraction, obtain by lysing the cellular pellet of *E. coli* BL21 (DE3) (New England Biolabs) cultured overnight, was loaded onto the prepared resin. Extensive wash was performed with MBP lysis buffer, and the protein was eluted with Elution Buffer (50 mM Glycine pH2.5), immediately buffered with Neutralization buffer (100 mM Tris-HCl pH 10). Aliquots of the samples collected from each purification step were treated with Laemmli Buffer and subjected to SDS-PAGE electrophoresis and Coomassie staining.

i. Fluorescent thermal shift assay and data analysis

The fluorescence-based thermal shift assay (Thermofluor) was performed using a StepOne Real-Time PCR System (Thermo Fisher Scientific, Waltham, MA, USA). A melting profile was generated using a linear temperature increase of 0.5 °C over a range of 25–95 °C. Reactions were performed in 25 µL final volume and assayed in 48-well plates. SYPRO Orange protein straining (5000X; Thermo Fisher Scientific, Waltham, MA, USA) was used at a final concentration of 8X (1:625 dilution). Recombinant MBP protein and the MBP–SdAbCM1 complex (1:1 molar ratio) were diluted in 1×

PBS (pH 7.4) to a final concentration of 2 μM for each protein. Negative derivative curves (-dRFU/dT) were calculated and the midpoint temperatures of the protein-unfolding transition (T_m; Figure S 2) were estimated using the StepOn Real-Time PCR System software (Thermofisher Scientific, Waltham, MA, USA). The effect of maltose binding on MBP, alone or in complex with sdAbCM1, was assessed by measuring its thermal stabilization effect at maltose concentrations ranging from 0 to 200 μM in isothermal conditions (Tm +1°C temperature). Mixtures of MBP, maltose, w/o sdAbCM1(1:1 stoichiometry) were incubated at room temperature for 30 min before the analysis. Control samples, including buffer and MBP protein only, were also analyzed. Briefly, EC_{50} was determined according to the unfolded fraction of MBP and MBP-sdAbCM1 at T_m+1 °C, plotted as a function of maltose concentrations and apparent K_D (K_D^{app}) values were calculated according to the model previously proposed (Bai et al., 2019). All the data were analysed using GraphPad Prism software (GraphPad Software, Boston, MA, USA).

j. Western blot

An increasing amount of pure MBP (0.025-1.5 μg), a total extract of *E. coli*, 20 μg of soluble fraction of HEK293 cells and a purified protein fused to MBP were loaded in SDS-PAGE. Proteins were transferred to 0.2 μm nitrocellulose membrane (Cytiva). Membrane was blocked with TBS-T with 5% (w/v) skimmed milk (TBST-M) for 1 hour, followed by overnight incubation with TBST-M and sdAbCM1 (50 μg/ml). The day after, membrane was rinsed with TBST and incubated with IgG anti-6His-tag HRP conjugated antibody diluted 1:20000 (SB194b, Southern Biotech). Bands were revealed with SuperSignal™ West Femto substrate (Thermofisher Scientific), using a Chemidoc Blot Imager (VWR).

k. Complex crystallization, data collection, structure determination and analysis

MBP – SdAbCM1 complex (1:1 molar ratio) was concentrated to 10 mg/ml and submitted to wide crystallization screenings in multiple conditions (Morpheus, LMB, PACT Premier and JCSG Plus crystallization kits, Molecular Dimensions) exploiting an Oryx8 Crystallization Robot (Douglas Instruments) in an isothermal vapor diffusion crystallization setup (0.6 μl total volume drops, 1:1 protein-precipitant ratio, 293 K). Best diffracting crystals were obtained with a precipitant buffer including 0.1 M MIB (Sodium malonate dibasic monohydrate, Imidazole, Boric acid) pH 7.0, 25 % w/v PEG 1500, and optimized by microseeding. Diffraction-quality crystals were soaked in the precipitant solution supplemented with 6% (w/v) D-Maltose and fast frozen in liquid nitrogen. Samples were stored in liquid nitrogen until the diffraction experiment and collected at 100 K.
Diffraction data were collected at beamline ID 30-A3 at ESRF Synchrotron Radiation Facility (Grenoble, France; data collection DOI: doi.org/10.15151/ESRF-ES-1119935153). Statistics and further details of the diffraction datasets are reported in Table S1. Crystals diffracted to 1.75 Å resolution. The structure was solved by advanced molecular replacement using Phaser software (Mccoy et al., 2007) and ccp4i2 platform (Winn et al., 2011). Multi component phasing methods were explored to solve the phase problem, using MBP protein model (PDB 1MPD) and an *in silico* model of SdAbCM1 generated by SwissModel (Waterhouse et al., 2018). Refinement of the obtained model was carried out by Refmac5 (Murshudov et al., 2011) and manual adjustment by the graphic software Coot (Emsley et al., 2010). According to the difference electron density maps, 387 water molecules and a maltose ligand molecule were clearly visible and added during the refinement steps. PyMol molecular graphics software (Schrodinger LLC) was used to generate illustrations. MBP – SdAbCM1 interface analysis was carried out by PISA and LigPlot web services at the European Bioinformatics Institute (Krissinel & Henrick, 2007; Laskowski et al., 2005). The final structure model and the experimental data have been deposited in the open-access Protein Data Bank with code 9TY2, and it has been assigned the following PDB DOI: https://doi.org/10.2210/pdb9TY2/pdb.

l. scAbCM1 competition MBP purification on amylose resin

100 µg of MBP were loaded on 50 µl of equilibrated in buffer A (20 mM Tris, 150 mM NaCl, 1 mM EDTA pH 8.0) amylose resin (NEB) either alone or precomplexed with sdAbCM1 in equimolar concentration.
Resin was then washed with buffer A and finally elution was performed with buffer A added with 10 mM maltose. Fractions for each chromatographic steps were collected and loaded on SDS-PAGE.

2. Supplementary figures

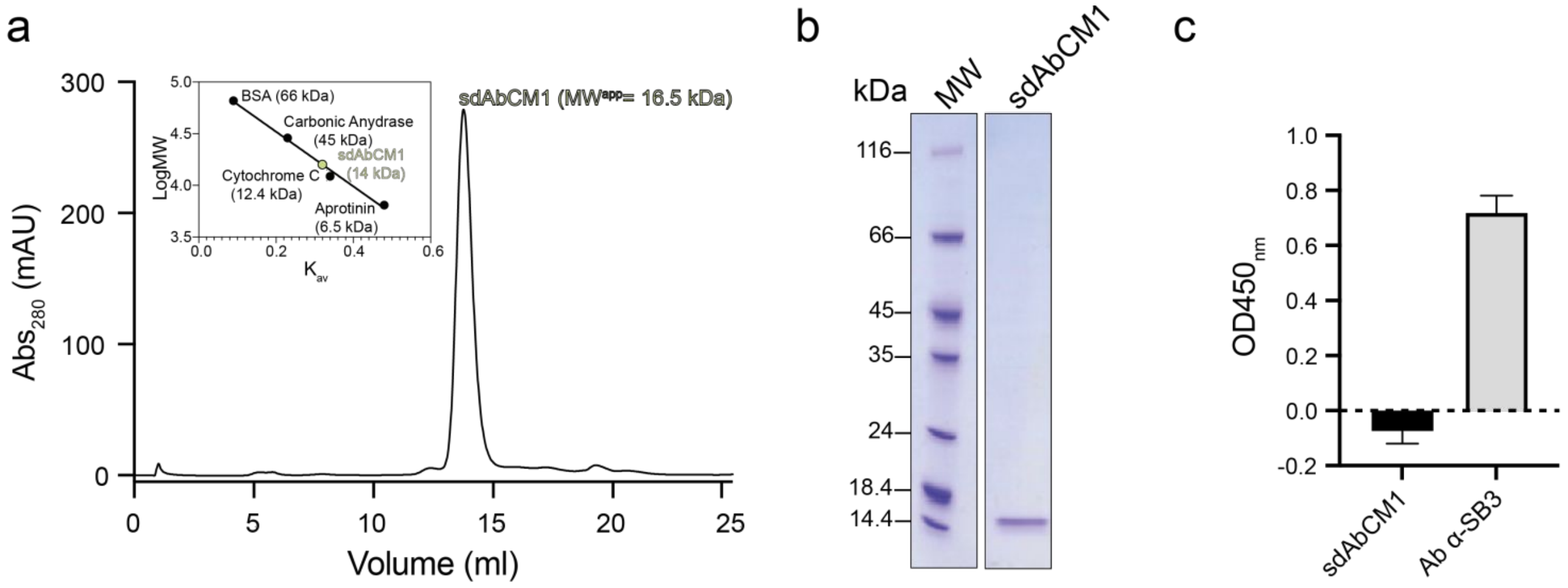

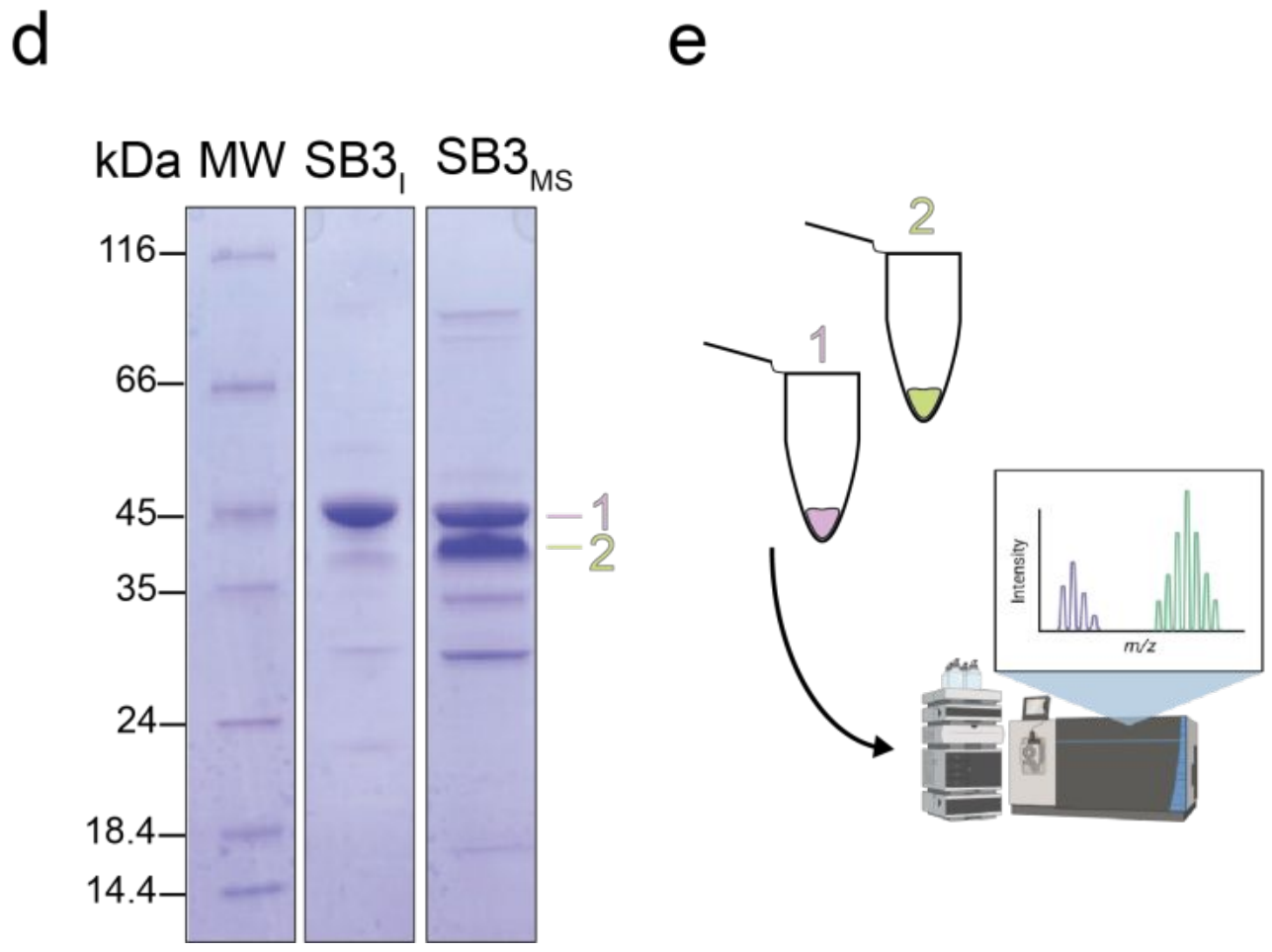

f

Sample ID	Peptide coverage (%)	Protein ID	Gene ID
1	90,5	P0AEX9	SERPINB3
2	44,2	P29508	MalE

Figure S1: Soluble sdAbCM1 validation and SB3 LC-MS/MS analysis and MBP identification. (a) Analytical size exclusion chromatography of sdAbCM1, with apparent molecular weight (MW^{app}) indicated, performed on Superdex75 10/300 resin (Cytiva); on the top-left comparison of sdAbCM1 with Protein Standards. (b) Coomassie stained SDS-PAGE of sdAbCM1 in comparison with MW lane. (c) ELISA assay results comparing sdAbCM1 response to 100 ng/ml of SERPINB3 compared with oligoclonal anti-SB3/4 antibody (Xeptagen, Venice, Italy). (d) Coomassie stained SDS-PAGE confronting SERPINB3 samples used for alpaca immunization ($SB3_I$) and the one used for LC-MS/MS analysis ($SB3_{MS}$), the two bands indicated as 1 and 2 were extracted from gel; molecular weights markers (MW) are reported in the first line of the gel (Unstained protein markers, Thermofisher Scientific). (e) Graphical representation of the band extracted from Coomassie SDS-PAGE, proteolyzed and subjected to LC-MS/MS. (f) Table with sample identification. The scheme representing the sample treatment and processing by mass-spectrometry was created using Microsoft PowerPoint and Biorender tools.

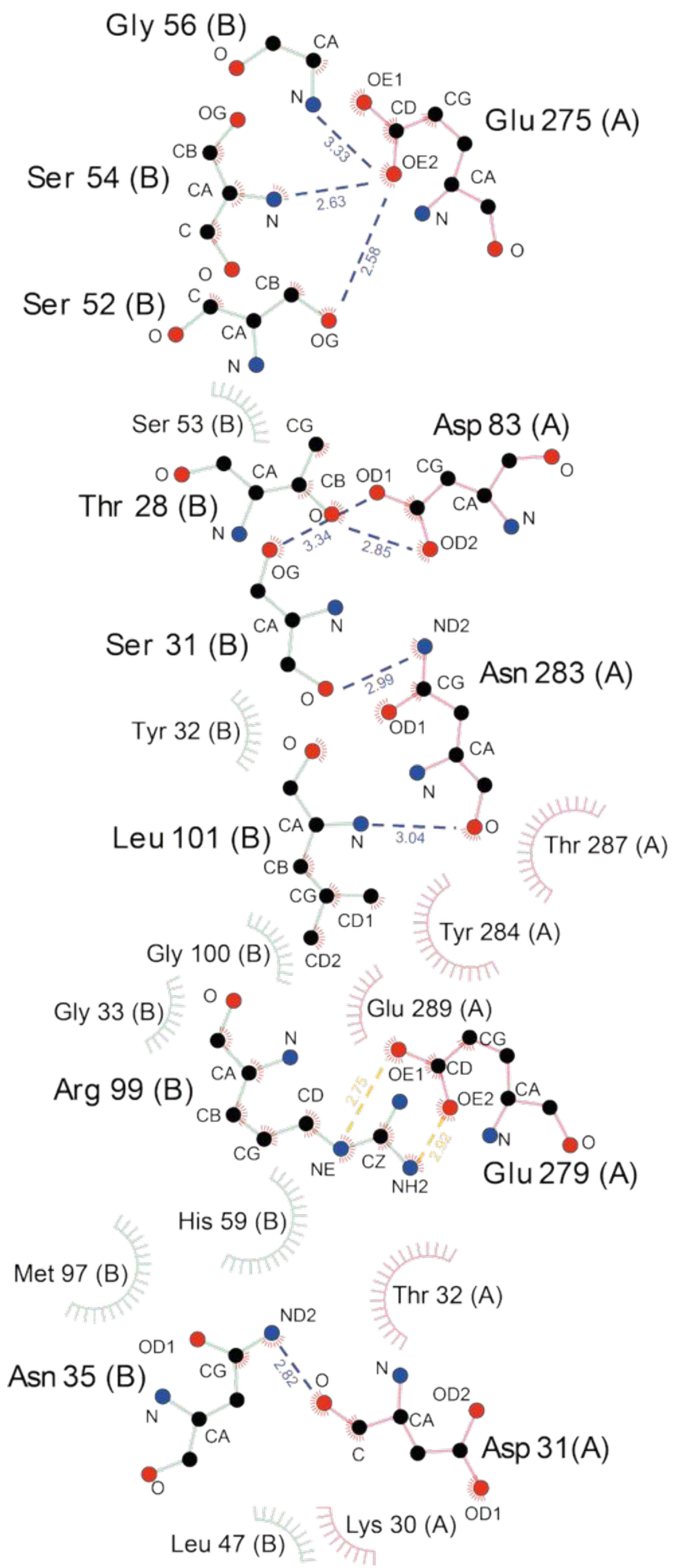

Figure S2: Ligplot map of MBP and sdAbCM1 interacting residues. Ligplot interaction map at sdAbCM1 and MBP interface. sdAbCM1 (chain B) residues are represented in green, while MBP (chain A) residues are presented in pink. Salt bridge between Arg99 of sdAbCM1 and Glu279 of MBP are presented in yellow dashed lines; hydrogen bonds are presented in blue dashed lines, while hydrophobic interactions are presented as spiked demi-circles around the residues involved.

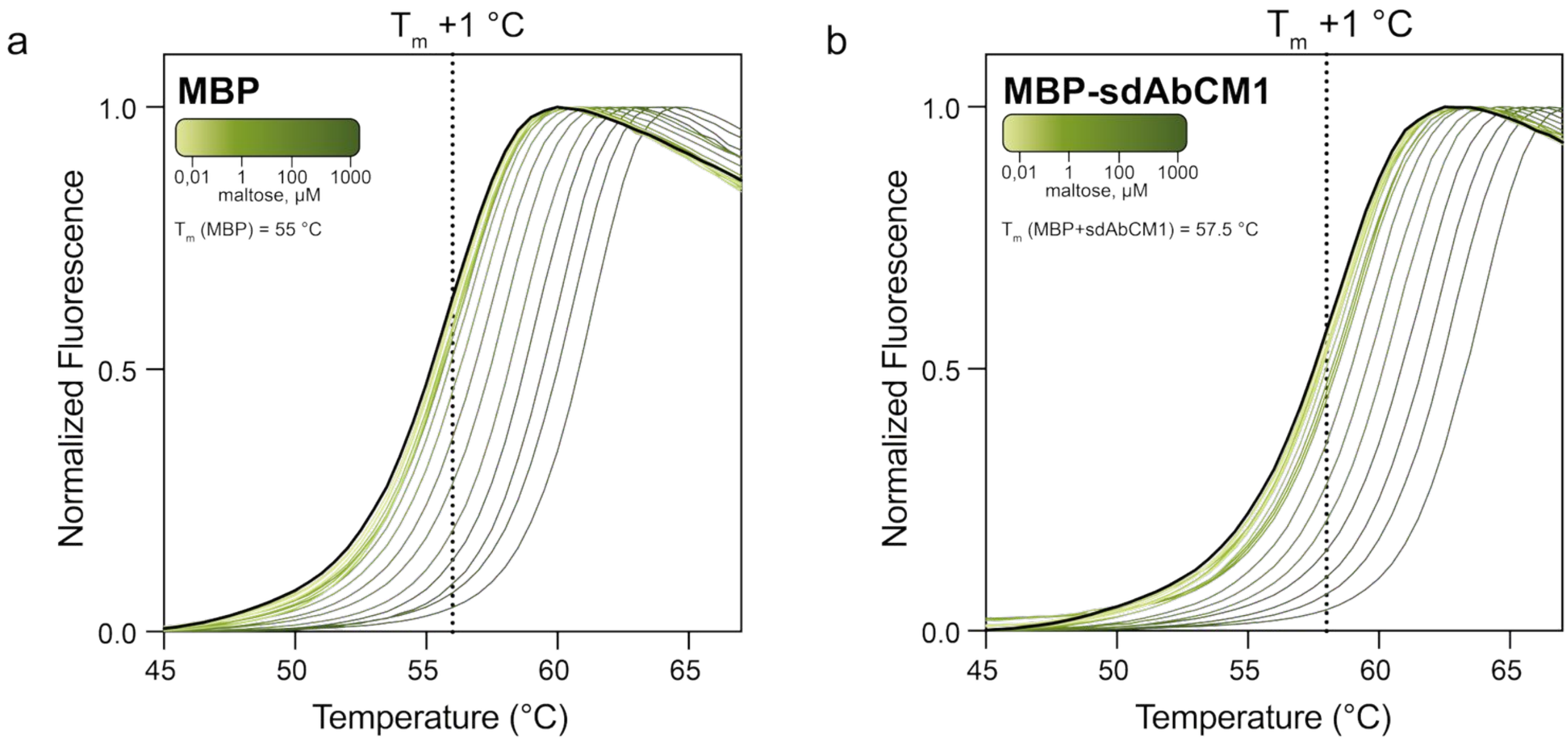

Figure S3: Normalized Thermofluor curves with maltose titration. (a) Denaturation curves of MBP in presence of increasing concentration of maltose. (b) Denaturation curves of MBP-SdAbCM1 complex in presence of increasing concentration of maltose.

In both cases the proteins' melting curve, MBP or MBP-sdAbCM1 complex, without ligand are represented in black and each respective experimental T_m is stated. Maltose concentrations are represented in shades of greens as graphically indicated in the color scale. The dotted line indicates the +1° C T_m of MBP and MBP stabilized by sdAbCM1 presence; these temperature values were chosen to extrapolate the unfolded fraction and estimate maltose EC_{50} and K_D^{app} in the presence or absence of sdAbCM1.

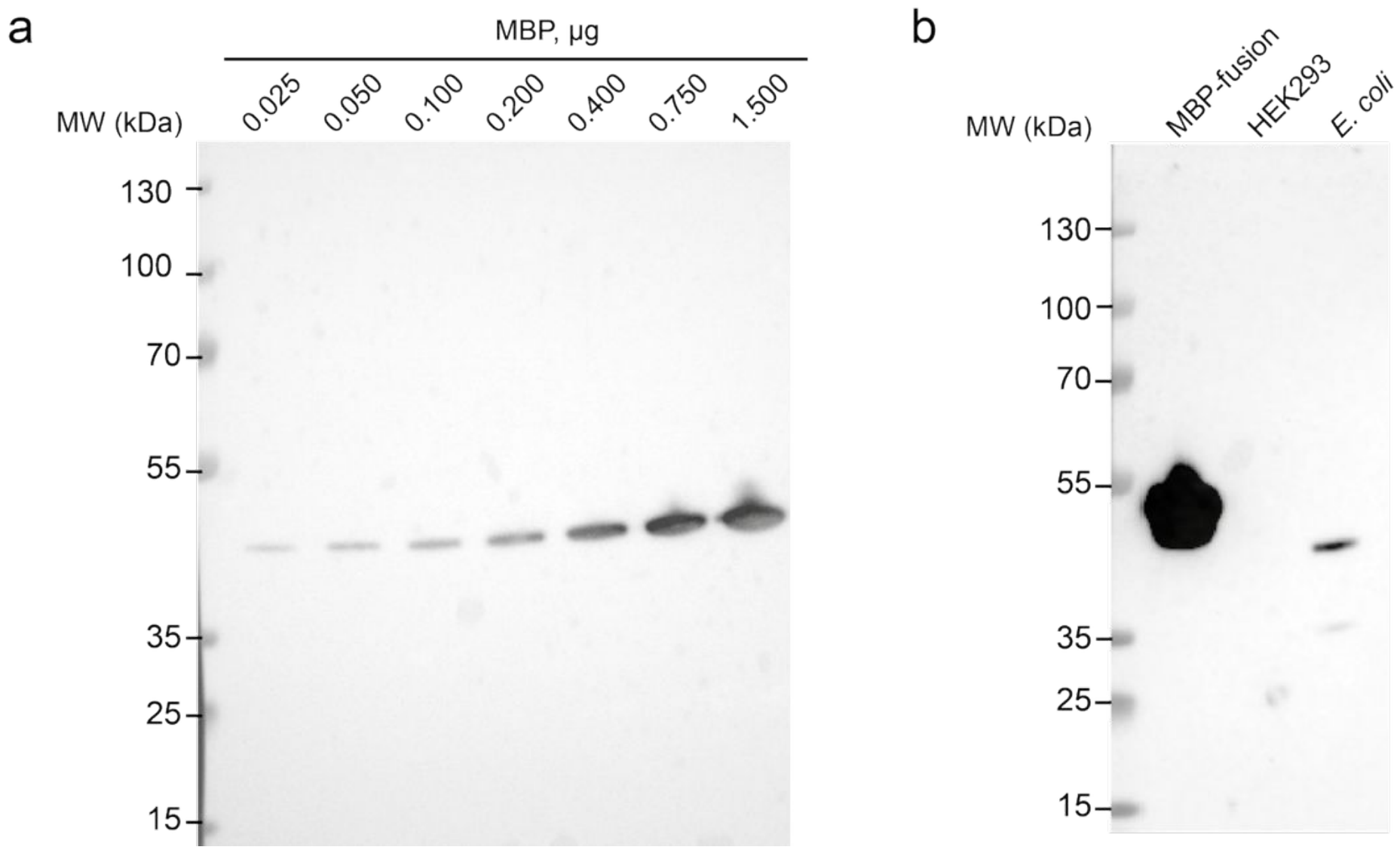

Figure S4: Detection on MBP. (a) Western blot detecting purified MBP ranging from 25 ng to 1.5 µg loaded in each lane. (b) Detection of MBP from 3 different samples: a purified protein fused to MBP, 20 µg of the soluble fraction of a HEK293 cells lysate and of *E.coli* crude lysate. In both cases primary detection was performed by incubation with sdAbCM1 50 µg/ml.

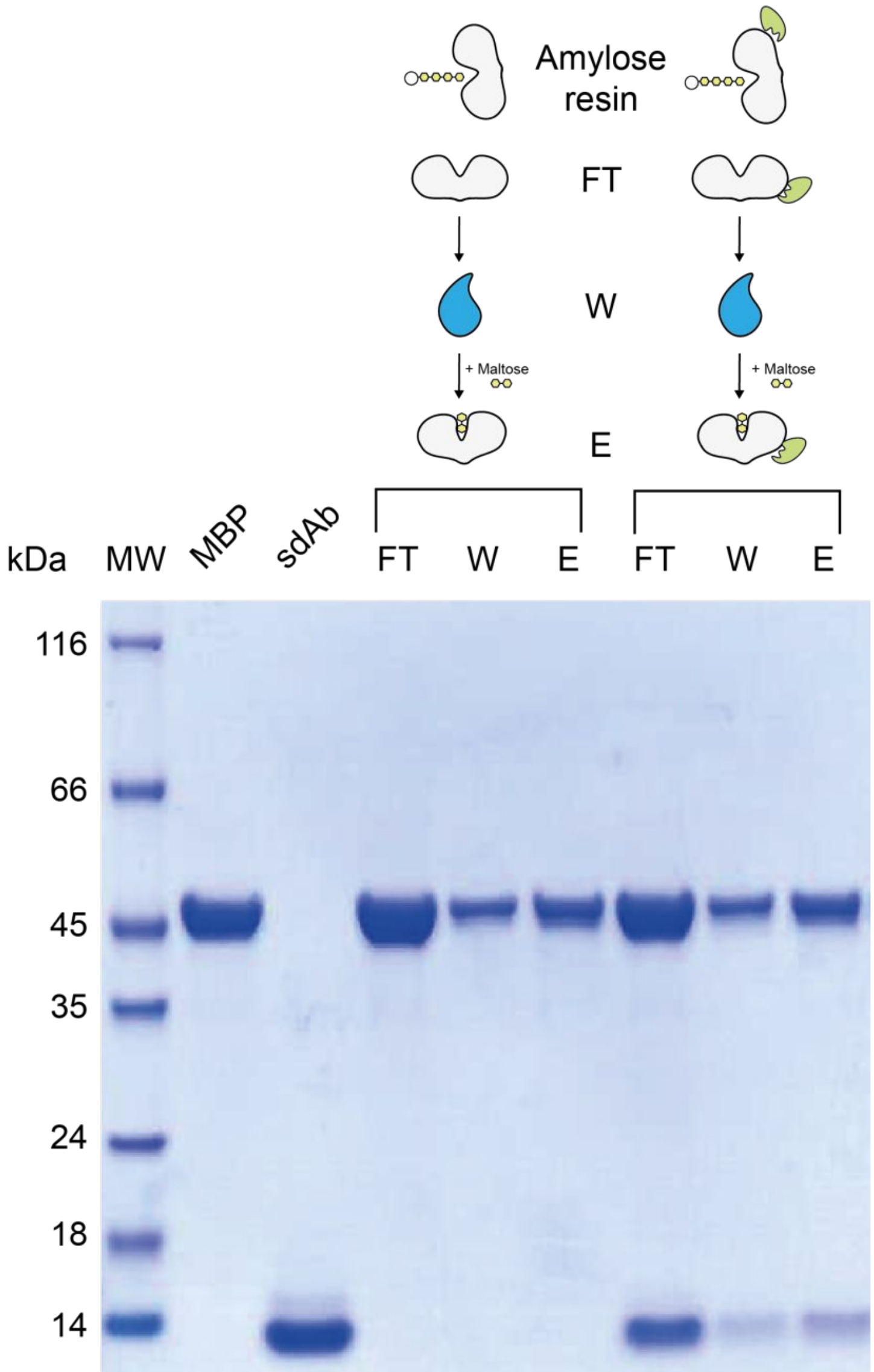

Figure S5: sdAbCM1 competition over MBP-amylose purification. Graphicals representing purification steps of the two distinct samples (MBP alone and MBP with sdAbCM1). Coomassie blue stained of a SDS-PAGE analysis comparing different fractions to MBP alone (MBP) and sdAbCM1 alone (sdAb). MW: molecular weight (Unstained protein markers, Thermofisher Scientific), FT: unbound fraction, W: wash, E: elution. The scheme representing the sample treatment and processing by mass-spectrometry was created using Microsoft PowerPoint and Biorender tools.

3. Supplementary Tables

Table S1. Nbs CDRs sequences with free cysteine in bold and highlighted in yellow.

ID	CDR1 (25-35)	CDR2 (50-60)	CDR3 (93-115)
SdAbCM1	SGFTFSSYGMNW	GISSSGGVTHY	YYCMR-----------RGLPAYW
NbCM2	SGFTFDDYAIAW	**C**ISPSDGVRYY	YYCAAAPGW**C**TEIYDRRYEYDYW
NbCM3	SGVSADNDAIGW	**C**ISISDDSTFY	YFCSVSYDA---LAWVGGHVNYW
NbCM4	SGYSLRNYAVGW	**C**ISSTDGSTYY	YYCATRKTYLY**C**SNYGGPDFGNW

Table S2. Proteins biochemical proteins estimated with Expasy ProtParam

Protein	Number of aminoacids	Molecular weight	Theorical pI
SERPINB3	390	44564.60	6.35
MBP	396	43387.64	5.53

Table S3. Crystallographic table

**Data collection**	
Diffraction source	ID30-A3 (ESRF)
Temperature (K)	100
Detector	Eiger_4M
Crystal-detector distance (mm)	99.88
Rotation range per image (°)	0.10
Exposure time per image (s)	0.005
Wavelength (Å)	0.968
Space group	$P1\ 2_1\ 1$
No. of molecules/ASU	1
Cell parameters	
a, b, c (Å); α, β, γ (°)	68.60 52.16 77.14, 90.000 92.075 90.000
Resolution (Å)	52.23-1.75 (1.78-1.75)
Total observations	206646 (11746)
Unique observations	54091 (2962)
Multiplicity	3.8 (4.0)
R_{merge}	0.164 (1.16)
$\langle I/\sigma(I) \rangle$	5.5 (0.8)
CC1/2	0.990 (0.40)
Completeness (%)	98.0 (98.3)
Wilson B-factor	25.4
**Refinement**	
No. reflections (used for R_{free} calculation)	54070 (2745)
R_{work} / R_{free}	0.19/0.23

Number non-hydrogen atoms	4199
protein (chains A, B)	3804
ligands (MAL)	23
solvent	371
**Geometry**	
RMSD values	
bond lengths (Å)	0.0099
bond angles (°)	1.24
Ramachandran plot (%)	
most favored	98.5
additionally allowed	1.5
outliers	0.00
Average B-factor	
protein (chains A, B)	28.67
ligands (MAL)	21.13
solvent	33.58
RSCC (MAL)	0.96

References:

Angelini, A., Chen, T. F., de Picciotto, S., Yang, N. J., Tzeng, A., Santos, M. S., Van Deventer, J. A., Traxlmayr, M. W., & and Wittrup, K. D. (2015). *Protein Engineering and Selection Using Yeast Surface Display*. Methods in Molecular Biology.

Bai, N., Roder, H., Dickson, A., & Karanicolas, J. (2019). Isothermal Analysis of thermoFluor Data can readily provide Quantitative Binding Affinities. *Scientific Reports*, *9*(2650). https://doi.org/10.1038/s41598-018-37072-x

Biasiolo, A., Sandre, M., Ferro, S., Quarta, S., Ruvoletto, M., Villano, G., Turato, C., Guido, M., Marin, O., & Pontisso, P. (2023). Epitope-Specific Anti-SerpinB3 Antibodies for SerpinB3 Recognition and Biological Activity Inhibition. *Biomolecules*, *13*(5), 739. https://doi.org/10.3390/BIOM13050739/S1

Emsley, P., Lohkamp, B., Scott, W. G., & Cowtan, K. (2010). Features and development of Coot. *Acta Crystallographica. Section D, Biological Crystallography*, *66*(Pt 4), 486–501. https://doi.org/10.1107/S0907444910007493

Krissinel, E., & Henrick, K. (2007). Inference of macromolecular assemblies from crystalline state. *Journal of Molecular Biology*, *372*(3), 774–797. https://doi.org/10.1016/J.JMB.2007.05.022

Laskowski, R. A., Watson, J. D., & Thornton, J. M. (2005). ProFunc: a server for predicting protein function from 3D structure. *Nucleic Acids Research*, *33*. https://doi.org/10.1093/nar/gki414

Mccoy, A. J., Grosse-Kunstleve, R. W., Adams, P. D., Winn, M. D., Storoni, L. C., & Read, R. J. (2007). Phaser crystallographic software. *J. Appl. Cryst*, *40*, 658–674. https://doi.org/10.1107/S0021889807021206

Murshudov, G. N., Skubák, P., Lebedev, A. A., Pannu, N. S., Steiner, R. A., Nicholls, R. A., Winn, M. D., Long, F., & Vagin, A. A. (2011). REFMAC5 for the refinement of macromolecular

crystal structures. *Acta Crystallographica. Section D, Biological Crystallography*, *67*(Pt 4), 355–367. https://doi.org/10.1107/S0907444911001314

Pardon, E., Laeremans, T., Triest, S., Rasmussen, S. G. F., Wohlkönig, A., Ruf, A., Muyldermans, S., Hol, W. G. J., Kobilka, B. K., & Steyaert, J. (2014). A general protocol for the generation of Nanobodies for structural biology. *Nature Protocols*, *9*(3), 674–693. https://doi.org/10.1038/nprot.2014.039

Schmidt, F. I., Lu, A., Chen, J. W., Ruan, J., Tang, C., Wu, H., & Ploegh, H. L. (2016). A single domain antibody fragment that recognizes the adaptor ASC defines the role of ASC domains in inflammasome assembly. *Journal of Experimental Medicine*, *213*(5), 771–790. https://doi.org/10.1084/JEM.20151790

Van Deventer, J. A., Kelly, R. L., Rajan, S., Wittrup, K. D., & Sidhu, S. S. (2015). *A switchable yeast display/secretion system*. https://doi.org/10.1093/protein/gzv043

Waterhouse, A., Bertoni, M., Bienert, S., Studer, G., Tauriello, G., Gumienny, R., Heer, F. T., De Beer, T. A. P., Rempfer, C., Bordoli, L., Lepore, R., & Schwede, T. (2018). SWISS-MODEL: homology modelling of protein structures and complexes. *Nucleic Acids Research*, *46*(W1), W296–W303. https://doi.org/10.1093/NAR/GKY427

Winn, M. D., Ballard, C. C., Cowtan, K. D., Dodson, E. J., Emsley, P., Evans, P. R., Keegan, R. M., Krissinel, E. B., Leslie, A. G. W., Mccoy, A., Mcnicholas, S. J., Murshudov, G. N., Pannu, N. S., Potterton, E. A., Powell, H. R., Read, R. J., Vagin, A., & Wilson, K. S. (2011). Biological Crystallography Overview of the CCP4 suite and current developments. *Research Papers Acta Cryst*, *67*, 235–242. https://doi.org/10.1107/S0907444910045749
